# Supplementary material for: Identification of Long Noncoding RNAs That Exert Transcriptional Regulation by Forming RNA–DNA Triplexes in Prostate Cancer
Source: Int J Mol Sci. 2023 Jan 19;24(3):2035. doi: 10.3390/ijms24032035 (PMC9916442; doi:10.3390/ijms24032035)
Supplement: Supplementary file 1 [file ijms-24-02035-s001.zip › Supplementary Materials.pdf]

Supplementary Materials for

Identification of long non-coding RNAs that exert transcriptional regulation by forming RNA-DNA triplexes in prostate cancer

Yugang Liang, Yali Lu, Qin Chen, Yihang Cheng, Yunsheng Ma, Yan Huang, Minyan Qiu and Yao Li

This PDF file includes:

Figure S1A. Kaplan-Meier survival curves of prostate patients with different overall survival gene expression levels.

Figure S1B. Kaplan-Meier survival curves of prostate patients with different disease-free survival gene expression levels.

Table S1. The differential expression of 5946 genes in cancer and normal tissues.

Table S2. 44,270 pairs of co-expression relationships Between 612 lncRNAs and 2,742 mRNAs.

Table S3.Data on 392 potential triplex-forming lncRNAs and target genes, and the number of R/Y/M-type PTS.

Table S4.Nine candidate lncRNAs according to the ranking of PTS scores and the other parameters.

Table S5.Co-expression of mRNAs with AD000684.2 and ASMTL-AS1 and their correlation coefficients.

Table S6. Primers used in DNA fragment generation.

Table S7. Nucleotide sequences used in triplex assays.

Table S8. siRNA sequences used in knockdown experiments.

Table S9. Primers used in qPCR.

Additional file S1. Raw data of 599 lncRNAs PTS

Additional file S2. Raw data of 392 lncRNA R-type TFO

Additional file S3. Raw data of 392 lncRNA Y-type TFO.

Additional file S4. Raw data of 392 lncRNA M-type TFO.

Supplementary Figures

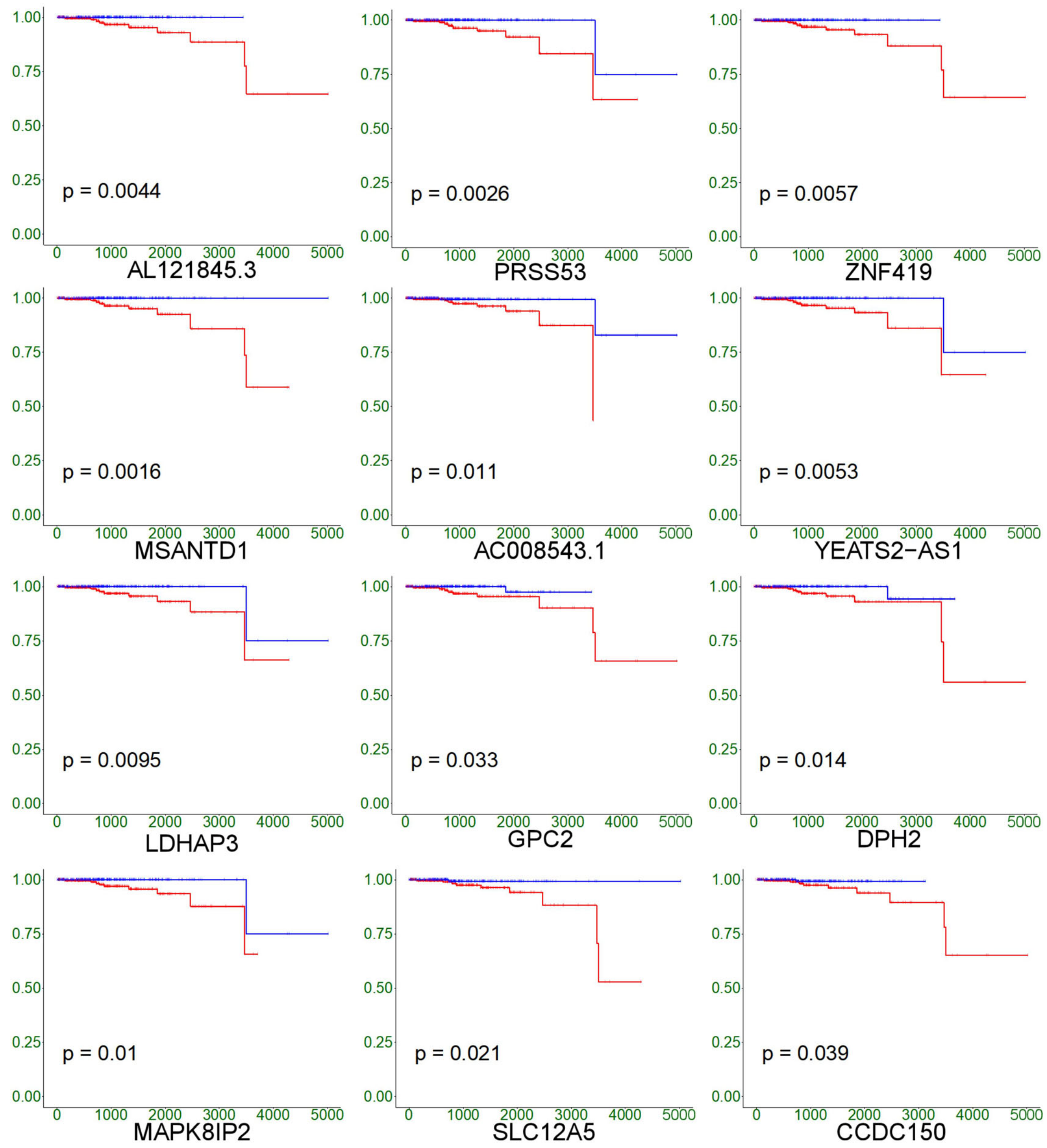

Figure S1A. Kaplan-Meier survival curves of prostate patients with different overall survival gene expression levels The x-coordinate represents time (in days), and the y-coordinate represents the overall survival rate;Blue For the survival rate of low-expression groups,Red For the survival rate of high-expression groups;p<0.05 indicated significant difference in survival rate between the two groups;the figure shows the Kaplan-Meier survival curve of prostate cancer patients with different expression levels of 12 overall survival genes.

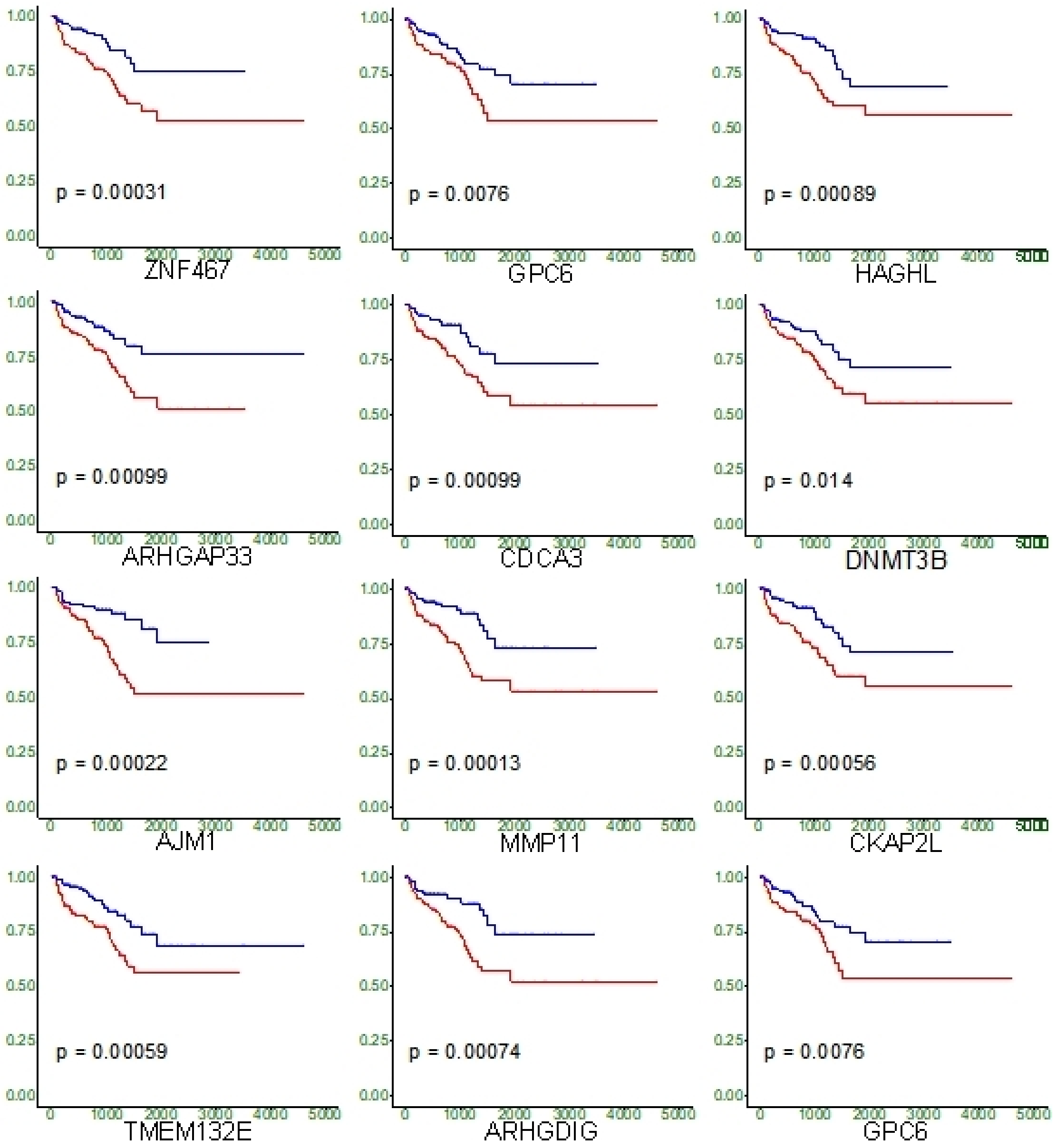

Figure S1B. Kaplan-Meier survival curves of prostate patients with different disease-free survival gene expression levels The x-coordinate represents time (in days), and the y-coordinate represents the overall survival rate;Blue For the survival rate of low-expression groups,Red For the survival rate of high-expression groups;$p<0.05$ indicated significant difference in survival rate between the two groups;the figure shows the Kaplan-Meier survival curve of prostate cancer patients with different expression levels of 12 disease-free survival genes.

Supplementary File

Table S1. The differential expression of 5946 genes in prostate cancer and normal tissues

The file contains GeneID, GeneType, symbol, logFC, FDR, RCI, OverallSurv, DiseaseFreeSurv information for 5946 genes. Among them, FDR indicates false discovery rate; Relative Concentration Index (RCI) indicates the logarithm of the ratio of concentration to nucleus (Log_2), positive value indicates distribution in cytoplasm, negative value indicates distribution in nucleus; 0 in OverallSurv and DiseaseFreeSurv indicates insignificant, 1 indicates significant.

Table S2.44,270 pairs of co-expression relationships Between 612 lncRNAs and 2,742 mRNAs

The file includes details of 44270 lncRNA-mRNA pairs, as well as information on 612 lncRNAs and 2742 mRNAs associated with 44274 co-expressed gene pairs. Where, r indicates the correlation coefficient between differentially expressed lncRNAs and mRNAs; p-values were corrected by the Benjamini-Hochberg algorithm to obtain FDR values.. $|r|>=0.6$ and FDR<0.01, between the lncRNA and mRNA,

Table S3.Data on 392 potential triplex-forming lncRNAs and target genes, and the number of R/Y/M-type.

The file includes information about 392 triplex lncRNA related to lncRNA.ensembl,lncRNA.symbol,Number of potential triplex target mRNA,percentage of positively correlated mRNA, as well as information about 7,839 purine motif (R),12,678 pyrimidine motif (Y), and 9,214 purine-pyrimidine motif (or mixed/M) PTS Information.

Table S4.Nine candidate lncRNAs according to the ranking of PTS scores and the other parameters.

The file includes logFC(log of fold change) of lncRNA expression, RCI, Log rank *p* of DFS, number of co-expression pairs and percentage of positively correlated mRNAs. as well as PTS scores of nine lncRNA.

Table S5.Co-expression of mRNAs with AD000684.2 and ASMTL-AS1 and their correlation coefficients.

The file consists of lncRNA AD000684.2 relevant 16 gene.ensembl,gene.symbol,r,FDR information,and lncRNA ASMTL-AS1 relevant 31 gene.ensembl,gene.symbol,r,FDR information.

Table S6. Primers used in DNA fragment generation.

Name	Sequence（5′ to 3′）
AD000684.2-T7-F	taatacgactcactatagggTAATACGACTCACTATAGGGAATGCTG
AD000684.2-T7-R	GCAAGTCTTTTTCAATTGCACATG

Table S7. Nucleotide sequences used in triplex assays.

Name	Sequence（5′ to 3′）
AD000684.2-TFO	AAGCAGGGGAAGGTAAGAGAAGACAGAAGACAGAGAGAGAGG
AD000684.2-TFO-mut	AAGCAGCGGTTGGTAACAGATCACAGAAGACAGTCAGACTGG
SLC23A3-FAM	GCAGAATGTTTTCTCTCCATCCTCTCCTCTCTTCTACCAGGTGTCCGGGG-TEG-FAM
CPT1B-FAM	GGGTTCTGAGCTAGACGGGAGAGGCAGACAGGGCACTGGTGCCTGGTGTG-TEG-FAM
MSH5-FAM	CTCCTATCTCAGTAGCTCCTCTTTCCCTCTCTGGGCTTCTCTTTCCACTC-TEG-FAM
KAT2A-FAM	TTCTATAGAGAGCACCCGAGAGAGAGAGGAGAAGGAGAACCTTGTCCCTG-TEG-FAM
LYG1-FAM	TTCCCCTTCTCACTCTCTCTCTCCTTCTCACTCTCTCTCTCTTTCTCTCT-TEG-FAM

Table S8. siRNA sequences used in knockdown experiments.

Name	Sequence（5′ to 3′）
si-NC	UUCUCCGAACGUGUCACGUTT
si-AD000684.2-1	GAGGGACCUAACUCUAUGATT
si-AD000684.2-2	GCCAAAUUUAAGCAAAUGUTT
si-ASMTL-AS1-1	GGGUCUUCCUGAAGUACAUTT
si-ASMTL-AS1-2	CUGGGUAAGAAGAGCUAAATT

Table S9. Primers used in qPCR.

Name	Sequence（5′ to 3′）
β-actin-F	CCTCTCCCAAGTCCACACAG
β-actin-R	GGGCACGAAGGCTCATCATT
AD000684.2-F	TCCCAACGGGACACTTTCAC
AD000684.2-R	CTACCCAGACACCCTCCTGA
ASMTL-AS1-F	ACGAGATACGTCCGTCAGGT
ASMTL-AS1-R	GGAGCTCTGTTCAAGTCCCC
SLC23A3-F	ACCAGAACGCCTACTTCTGC
SLC23A3-R	GCAACCCACCCTATTGGGAA
CPT1B-F	TGGGCTTACCTAGAGTCTGTG
CPT1B-R	TCCCACCAGTCACTCACATAG
MSH5-F	GAAGATAAGCGCGTGAGGCT
MSH5-R	ATGAGCTTGGAGGCTCCTTTG
KAT2A-F	CCCGCTACGAAACCACTCAT
KAT2A-R	GCATGGACAGGAATTTGGGGA
LYG1-F	TGGTCAACATGGGCGATAGG
LYG1-R	TCTCAGGTACTGGTCAGGGG
PRR22-F	GCACCCCAAACCGTTCTGT
PRR22-R	ATACAGGTTCAAGGAGCCCAT
RGL2-F	GCAGTCCTTTCTGAGTTGCG
RGL2-R	CACAGGATACACGATGGCTCT

Additional file S1. Raw data of 599 lncRNAs PTS.

The file consists of 599 lncRNA PTS, rendered in tfo format.

Additional file S2. Raw data of 392 lncRNA R-type TFO

The file consists of 3377 R-type TFO information predicted by Triplexator, rendered in tpx format.

Additional file S3. Raw data of 392 lncRNA Y-type TFO

The file consists of 3537 Y-type TFO information predicted by Triplexator, rendered in tpx format.

Additional file S4. Raw data of 392 lncRNA M-type TFO

The file consists of 2309 M-type TFO information predicted by Triplexator, rendered in tpx format.
