# Supplementary material for: Identification of Long Noncoding RNAs That Exert Transcriptional Regulation by Forming RNA–DNA Triplexes in Prostate Cancer
Source: Int J Mol Sci. 2023 Jan 19;24(3):2035. doi: 10.3390/ijms24032035 (PMC9916442; doi:10.3390/ijms24032035)
Supplement: Supplementary file 1 [file ijms-24-02035-s001.zip › table S7.pdf]

Table S7. Nucleotide sequences used in triplex assays.

Name	Sequence（5′ to 3′）
AD000684.2-TFO	AAGCAGGGGAAGGTAAGAGAAGACAGAAGACA GAGAGAGAGG
AD000684.2-TFO-mut	AAGCAGCGGTTGGTAACAGATCACAGAAGACAG TCAGACTGG
SLC23A3-FAM	GCAGAATGTTTTCTCTCCATCCTCTCCTCTCTTC TACCAGGTGTCCGGGG-TEG-FAM
CPT1B-FAM	GGGTTCTGAGCTAGACGGGAGAGGCAGACAGG GCACTGGTGCCTGGTGTG-TEG-FAM
MSH5-FAM	CTCCTATCTCAGTAGCTCCTCTTTCCCTCTCTGG GCTTCTCTTTCCACTC-TEG-FAM
KAT2A-FAM	TTCTATAGAGAGCACCCGAGAGAGAGAGGAGAA GGAGAACCTTGTCCCTG-TEG-FAM
LYG1-FAM	TTCCCCTTCTCACTCTCTCTCTCCTTCTCACTCT CTCTCTCTTTCTCTCT-TEG-FAM
