# Supplementary material for: Identification of Long Noncoding RNAs That Exert Transcriptional Regulation by Forming RNA–DNA Triplexes in Prostate Cancer
Source: Int J Mol Sci. 2023 Jan 19;24(3):2035. doi: 10.3390/ijms24032035 (PMC9916442; doi:10.3390/ijms24032035)
Supplement: Supplementary file 1 [file ijms-24-02035-s001.zip › table S9.pdf]

Table S9. Primers used in qPCR.

Name	Sequence（5′ to 3′）
β-actin-F	CCTCTCCCAAGTCCACACAG
β-actin-R	GGGCACGAAGGCTCATCATT
AD000684.2-F	TCCCAACGGGACACTTTCAC
AD000684.2-R	CTACCCAGACACCCTCCTGA
ASMTL-AS1-F	ACGAGATACGTCCGTCAGGT
ASMTL-AS1-R	GGAGCTCTGTTCAAGTCCCC
SLC23A3-F	ACCAGAACGCCTACTTCTGC
SLC23A3-R	GCAACCCACCCTATTGGGAA
CPT1B-F	TGGGCTTACCTAGAGTCTGTG
CPT1B-R	TCCCACCAGTCACTCACATAG
MSH5-F	GAAGATAAGCGCGTGAGGCT
MSH5-R	ATGAGCTTGGAGGCTCCTTTG
KAT2A-F	CCCGCTACGAAACCACTCAT
KAT2A-R	GCATGGACAGGAATTTGGGGA
LYG1-F	TGGTCAACATGGGCGATAGG
LYG1-R	TCTCAGGTACTGGTCAGGGG
PRR22-F	GCACCCCAAACCGTTCTGT
PRR22-R	ATACAGGTTCAAGGAGCCCAT
RGL2-F	GCAGTCCTTTCTGAGTTGCG
RGL2-R	CACAGGATACACGATGGCTCT
