# Supplementary material for: Identification of Long Noncoding RNAs That Exert Transcriptional Regulation by Forming RNA–DNA Triplexes in Prostate Cancer
Source: Int J Mol Sci. 2023 Jan 19;24(3):2035. doi: 10.3390/ijms24032035 (PMC9916442; doi:10.3390/ijms24032035)
Supplement: Supplementary file 1 [file ijms-24-02035-s001.zip › table S6.pdf]

Table S6. Primers used in DNA fragment generation.

Name	Sequence（5′ to 3′）
AD000684.2-T7-F	taatacgactcactatagggTAATACGACTCACTATAGGGAATGCTG
AD000684.2-T7-R	GCAAGTCTTTTTCAATTGCACATG
